# Gut microbiome function and composition in infants from rural Kenya and association with human milk oligosaccharides

**DOI:** 10.1080/19490976.2023.2178793

**Published:** 2023-02-16

**Authors:** Muriel Derrien, Nadja Mikulic, Mary A Uyoga, Empar Chenoll, Eric Climent, Adrian Howard-Varona, Suzane Nyilima, Nicole U Stoffel, Simon Karanja, Robert Kottler, Bernd Stahl, Michael B Zimmermann, Raphaëlle Bourdet-Sicard

**Affiliations:** a Advanced Health & Science, Danone Nutricia Research, Palaiseau, France; b Laboratory of Human Nutrition, Department of Health Sciences and Technology, ETH Zurich, Switzerland; c ADM-Biopolis, ADM, Parc Cientific Universitat de Valencia, Paterna, Valencia, Spain; d Public and Community Health Department, Jomo Kenyatta University of Agriculture and Technology, Nairobi, Kenya; e glyXera GmbH, Magdeburg, Germany; f Advanced Health & Science, Danone Nutricia Research, Utrecht, The Netherlands; g Department of Chemical Biology & Drug Discovery, Utrecht Institute for Pharmaceutical Sciences, Utrecht University, Utrecht, The Netherlands

**Keywords:** Kenyan infants, gut microbiome, breastfeeding, *bifidobacterium*, community types

## Abstract

The gut microbiota evolves rapidly after birth, responding dynamically to environmental factors and playing a key role in short- and long-term health. Lifestyle and rurality have been shown to contribute to differences in the gut microbiome, including *Bifidobacterium* levels, between infants. We studied the composition, function and variability of the gut microbiomes of 6- to 11-month-old Kenyan infants (*n* = 105). Shotgun metagenomics showed *Bifidobacterium longum* to be the dominant species. A pangenomic analysis of *B. longum* in gut metagenomes revealed a high prevalence of *B. longum* subsp. *infantis* (*B. infantis*) in Kenyan infants (80%), and possible co-existence of this subspecies with *B. longum* subsp. *longum*. Stratification of the gut microbiome into community (GMC) types revealed differences in composition and functional features. GMC types with a higher prevalence of *B. infantis* and abundance of *B. breve* also had a lower pH and a lower abundance of genes encoding pathogenic features. An analysis of human milk oligosaccharides (HMOs) classified the human milk (HM) samples into four groups defined on the basis of secretor and Lewis polymorphisms revealed a higher prevalence of HM group III (*Se+, Le*-) (22%) than in most previously studied populations, with an enrichment in 2′-fucosyllactose. Our results show that the gut microbiome of partially breastfed Kenyan infants over the age of six months is enriched in bacteria from the *Bifidobacterium* community, including *B. infantis*, and that the high prevalence of a specific HM group may indicate a specific HMO-gut microbiome association. This study sheds light on gut microbiome variation in an understudied population with limited exposure to modern microbiome-altering factors.

## Introduction

The gut microbiota evolves rapidly after birth in response to host and environmental factors. Its composition and function affect short- and long-term health.^1^ Increasing numbers of studies monitoring the dynamics of gut microbiota establishment and maturation during the first few years of life and their effects on health and disease across populations are revealing both common global patterns of gut microbiota development,^2,3^ and distinctive features.^4,5^ The gut microbiome of infants living in rural areas and with low rates of immune disorders has consistently been shown to be enriched in *Bifidobacterium*, and, specifically, in *B. longum* subsp. *infantis* (*B. infantis*)^6–10^ relative to that of infants living in more urbanized or industrial settings.^8,10–12^ These findings were recently borne out by a global meta-analysis of 1900 fecal samples from healthy infants from 18 populations with different lifestyles.^13^ There are growing clinical evidence to support an association between low levels of *Bifidobacterium* and the prevalence of chronic and autoimmune diseases (reviewed by)^14^ and systemic inflammation.^15^ In resource-poor countries, infants are exposed to different environmental factors, the rate of vaginal delivery is higher and infants are breastfed for longer periods. Human milk (HM) has multiple nutritional and immunological benefits and is associated with both short- and long-term health benefits, and with a lower risk of developing several chronic diseases later in life (reviewed by).^16^ HM contains various bioactive compounds, including secretory IgA, antimicrobial factors, and human milk oligosaccharides (HMOs), which can be metabolized by various gut bacteria, including *Bifidobacterium* in particular (reviewed by).^17^ The HMO profile of HM is highly variable and affected by multiple factors, including geographic location, lactation stage, and genetics.^18^ Genetic factors make the largest contribution to the high variability of HMO profile, which depends on the maternal Secretor (*Se*) and Lewis (*Le*) genes. Mothers with a functional α-1-2-fucosyltransferase (FUT2) are described as having the secretor phenotype. They produce milk that contains HMOs, with residual amounts of α-1-2-fucose (reviewed elsewhere).^19,20^ Most mothers in diverse populations have the secretor phenotype.^21^ The reported association between secretor status and the infant gut microbiome varies between studies.^22–28^ Mothers with a functional α1-3/4-fucosyltransferase gene (FUT3) are referred to as “Lewis-positive”.^18^ FUT2/FUT3 polymorphisms result in four different milk groups: HM group I (Se^+^,Le^+^), HM group II (Se^–^, Le^+^), HM group III (Se^+^, Le^–^), and HM group IV (Se^–^, Le^–^), with HM groups I and II typically accounting for > 80% of mothers across different populations (reviewed by).^29^ However, fewer studies have investigated FUT2/FUT3 polymorphism and its association with the gut microbiome in African populations.^28,30^ Previous studies have shown that the gut microbiota of Kenyan infants breastfed for six to nine months and given supplementary food is dominated by *Bifidobacterium*.^28,31,32^ However, the taxonomic resolution of these studies was limited, and no functional assessment of the gut microbiome was performed. We conducted a single-blind randomized study in infants from rural Kenya, to evaluate the effects on iron absorption, inflammation and fecal microbiota composition of an iron-fortified wheat-based cereal (NCT03894358) containing two different doses of a prebiotic mixture including short chain galacto-oligosaccharides (GOS) and long chain fructo-oligosaccharides (FOS) (9:1 ratio). Here, we describe the use of shotgun metagenomics to assess the variability of the gut microbiome at baseline in a subset of these Kenyan infants (105 infants, 6 to 11 months postpartum).

## Results

### *Gut microbiome of infants from rural* Kenya

**Figure 1.**
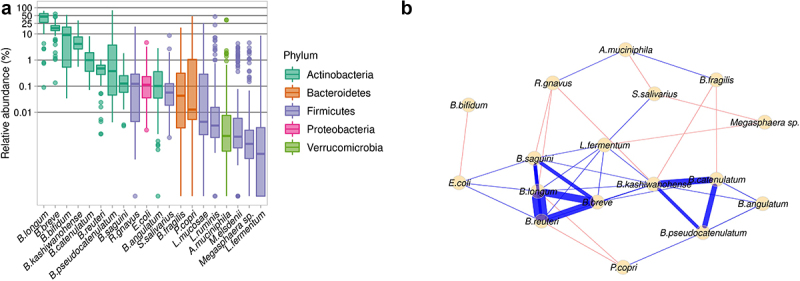
Gut microbiome of the infants from rural Kenya included in the study cohort. A. Abundance of bacterial species in the study cohort. B. Network based on the 20 most abundant species. Blue edges correspond to positive estimated associations; Red edges correspond to negative estimated associations and the thickness of the lines indicates the strength of the correlation between taxa.

We included 105 infants aged 8.31 ± 1.38 months (mean ± SD) and with a weight-for-age *z*-score of −0.43 ± 1.23 in this study. All infants received complementary foods (starting at the age of 5.72 ± 1.05 months), mostly in the form of maize porridge, and 104 infants were still partially breastfed at the time of the study. The gut microbiome was profiled by shotgun metagenomics, its composition was determined, and a functional analysis was performed (Figure S1). Species-level analysis showed that *B. longum* was the most abundant species (42.67 ± 20.10%), followed by *B. breve* (17.30 ± 8.52%), *B. bifidum* (12.82 ± 13.09%), and *B. kashiwanohense* (5.94 ± 5.02%) (Figure 1a and Table S1). Network analysis was performed by CLR normalization and SparCC correlation based on the 20 most abundant species. Two subnetworks (i.e. species highly correlated with each other) of *Bifidobacterium* species were identified. One subnetwork was based on a positive association between *B. kashiwanohense, B. catenulatum, B. pseudocatenulatum*, and *B. angulatum*. The other was based on a positive association between *B. breve, B. longum*, and *B. reuteri*. The species with the largest number of connections and associations was *B. kashiwanohense*, followed by *B. longum* (Figure 1b). The gut microbiome of partially breastfed infants from rural Kenyan was, thus, enriched in *Bifidobacterium*, with specific connections between species.

### *Pangenomic analysis of B.* *longum in the gut microbiome of infants from rural* Kenya

**Figure 2.**
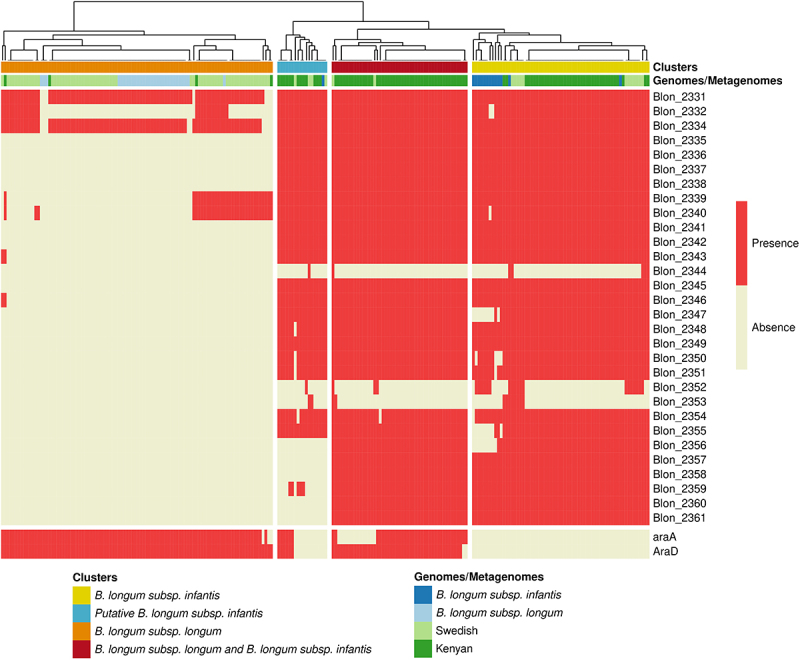
Identification of *B. longum* subsp. *longum* and *B. longum* subsp. *infantis* in metagenomes. Detection of genes related to *B. longum* subsp *infantis* (HMO clusters) and *B. longum* subsp. *longum* (*araD* and *araA*) in metagenomes from rural Kenyan and Swedish infants (dark and light green, respectively) and in reference genomes of *B. longum* subsp. *longum* and *B. infantis*. Clusters indicate the resulting stratification of metagenomes with the exclusive detection of *B. infantis* (yellow and blue) and *B. longum* subsp. *longum* (Orange) or the detection of both *B. infantis* and *B. longum* subsp. *longum* (red); clustering was performed by complete linkage analysis (Euclidean distance).

Given the high abundance of the species *B. longum* in our study cohort, and previous studies showing a high prevalence of *B. longum* subsp. *infantis* (*B. infantis*) in infants from non-industrialized countries,^13^ we assessed the prevalence of *B. infantis* in our study cohort. We used a strain-specific metagenomic approach on the pangenome of species *B. longum* with PanPhlAn (Figure 2 and Table S2). We focused on a cluster of genes encoding enzymes relating to HMOs import and metabolism (Blon_2331–Blon_2361) that is part of the larger H-1 cluster shown to be conserved in *B. infantis* genomes^33^ and two genes encoding proteins involved in arabinose consumption (*araA* and *araD)* used for the identification of *B. longum* subsp. *longum* in a previous study.^34^ Most of *B. infantis* genomes harbored almost all the genes from the HMO cluster. The *araA* and *araD* genes were detected in all the *B. longum* subsp. *longum* genomes and none of the *B. infantis* genomes included in the study. We then compared the metagenomes from our study cohort with 98 metagenomes from four-month-old infants from a Swedish cohort^35^ (Figure 2). In this Swedish cohort, 85% of infants were vaginally delivered and 68.8% of infants were exclusively breast-fed, 19.8% mixed fed, and 11.4% exclusively formula fed. *B. longum* was the major *Bifidobacterium* species, but its abundance was lower (21.23 ± 24.63%) than that in the infants from rural Kenya. A first set of metagenomic samples (39% from Kenyan infants and 15% from Swedish infants) clustered together with *B. infantis* genomes with positive detection of the HMO cluster and no detection of *araA* and *araD*. This cluster was considered to correspond exclusively to *B. infantis*. A second set of samples (12% of metagenomes from Kenyan infants and 5% from Swedish infants) displayed partial HMO cluster detection, mostly with an absence of *araA* and *araD*, and was considered to be a putative *B. infantis* group. A third set of samples (46% of Kenyan infants and 2% of Swedish infants) had both a detectable HMO cluster and detectable *araA* and *araD* genes, consistent with the co-existence of the two *B. longum* subspecies, whereas 4% of samples from Kenyan infants and 78% of those from Swedish infants clustered together with *B. longum* subsp. *longum* genomes, lacked the HMO cluster and had detectable *araA* and *araD* genes. This last cluster was considered to correspond exclusively to *B. longum* subsp. *longum*. A previous studies reported a lack of detection of genes Blon 2175_2177 (LNT transporter) in *B. infantis* from Bangladeshi infants.^36^ We assessed whether these genes were detected in metagenomes of Kenyan infants. We found that 80% of metagenomes that harbored Blon 2331–2361 had at least two genes of Blon 2175_2177 (Table S2). Overall, our pangenomic analysis, showed that *B. infantis* was highly prevalent in the gut microbiome of partially breastfed infants from rural Kenya.

### *Gut microbiome community types in infants from rural* Kenya

**Figure 3.**
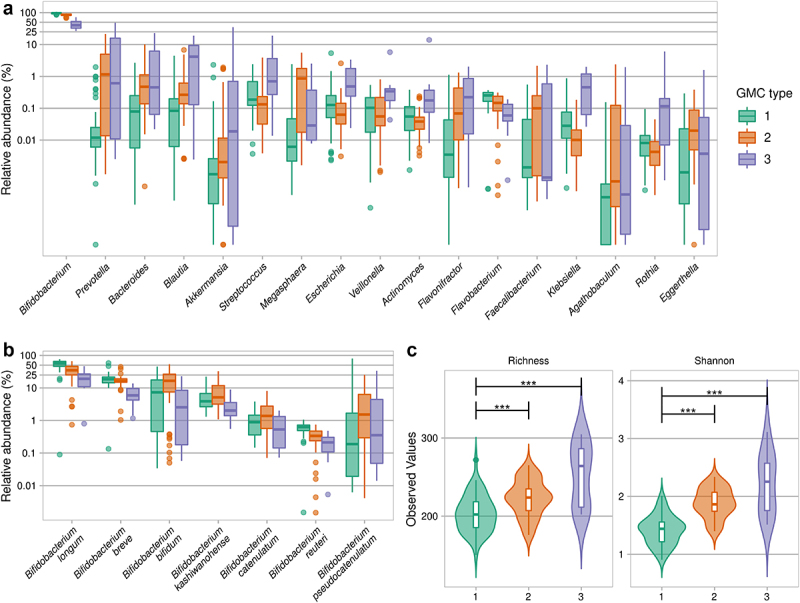
Gut microbiota community types in infants from rural Kenya. A. Abundances of the highest ranked bacterial genera that were statistically significantly different between the three GMC types. B. Abundance of significant *Bifidobacterium* species (Kruskal-Wallis test, followed by a post-hoc Dunn test; for details see Table S3 for panels A and B). C. Species-based alpha-diversity (species richness and Shannon index). ***p < .001 (Kruskal-Wallis test, followed by a post-hoc Dunn test).

We further explored gut microbiota variation between infants by clustering-based approaches. Dirichlet multinomial mixtures (DMM) based on genus relative abundance, as previously described,^37,38^ revealed that the infant gut microbiotas could be split optimally into three to four gut microbiome community (GMC) types (Laplace) (Figure S2), three of which were retained for further characterization to increase statistical power. GMC type 1 (50.5% of infants) had the highest levels of *Bifidobacterium* (Kruskal-Wallis test followed by a post-hoc Dunn test, FDR <0.001) (Figure 3a, Table S1 and Table S3), particularly for *B. longum*, the abundance of which decreased with increasing GMC type number (53.38 ± 16.10% for GMC type 1, 34.12 ± 17.61% for GMC type 2, and 19.91 ± 12.93% for GMC type 3) (Figure 3b, Table S1). GMC type 2 (40.0% of infants) was also enriched in *Bifidobacterium*, specifically *B. bifidum* (Kruskal-Wallis test followed by a post-hoc Dunn test, FDR <0.05), and was the GMC type most enriched in *Megasphaera elsdenii* (Figure S3A, Table S1 and Table S3). GMC type 3 (9.5% of infants) was the most enriched in various other genera, including those related to Enterobacteriaceae *Escherichia* and *Klebsiella*, and had the lowest abundance of *Bifidobacterium* (44.10 ± 16.09% (Table S1), notably *B. breve* (Table S3). Alpha-diversity differed between GMC types, with the lowest values obtained for GMC type 1 (Figure 3c).

**Figure 4.**
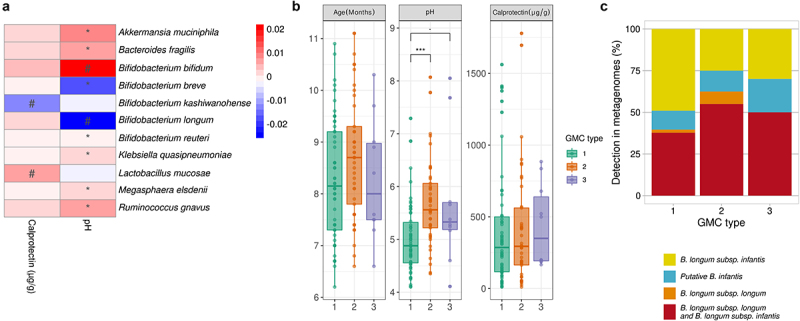
Association between host microbiota markers and the gut microbiota. A. Heatmap of the model coefficient values resulting from the MaAsLin2 analysis of the top 20 species, fecal calprotectin levels and pH. * indicates FDR<0.1 ^#^ indicates *p*< .1. B. Age, pH and calprotectin level distributions between GMC types (Kruskal-Wallis test, followed by a post-hoc Dunn test). “.”*p*= .05, *** *p* < .001. C. Exclusive presence of *B. infantis* or *B. longum* subsp. *longum* or the presence of both species in the three GMC types.

Linear mixed models implemented in MaAsLin2 (adjusted for subject and age) for the relationships between the most abundant bacterial species (accounting for 93.84 ± 6.19% of the gut microbiota) and pH revealed positive correlations for *A. muciniphila, B. fragilis, R. gnavus*, and *Klebsiella quasipneumoniae* and a negative correlation for *B. breve* (FDR <0.1). The abundances of *B. longum* and *B. kashiwanohense* were negatively related to pH (*p*= .06) and calprotectin levels (*p*= .008) respectively, before but not after FDR adjustment (FDR = 0.14–0.16) (Figure 4a and Table S4). Fecal pH varied with GMC type (Kruskal-Wallis *p*< .001) with a lower pH for GMC type 1 (5.01 ± 0.65) than for GMC type 2 (5.70 ± 0.81) and GMC type 3 (5.68 ± 1.24) (*p*< .001 and *p*= .05, respectively, in post-hoc Dunn tests) (Figure 4b). Age and calprotectin levels did not differ significantly between GMC types (Kruskal-Wallis *p*= .17 and 0.62, respectively) (Figure 4b). Given previous reports of the association between low pH and the presence of *B. infantis*,^39^ we further investigated whether the detection of the HMO cluster only (*B. infantis)* differed between the GMC types. We found that GMC type 1, for which the lowest pH values were recorded, had a higher percentage of metagenomes corresponding exclusively to *B. infantis* (60%) than the other types (Figure 4c).

**Figure 5.**
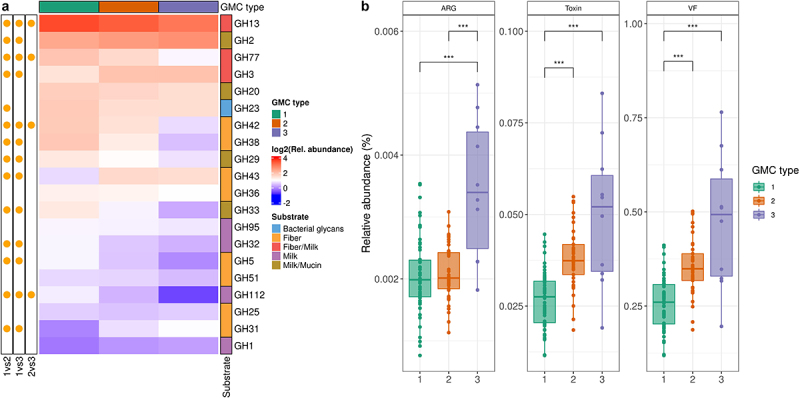
Functional assessment of gut microbiota in the three GMC types. A. Heatmap of the most abundant glycoside hydrolases (GH). Significance (on the left) of differences between GMC types (log_2_ of mean relative abundance) (Kruskal-Wallis test, followed by a post-hoc Dunn test; FDR<0.05). GHs are ordered in decreasing abundance. The substrates for GHs were taken from Qin et al^40^ B. Relative abundances of antimicrobial resistance genes (ARG), toxins and virulence factors (VF), as predicted by PathoFact (* *p* < .05, ** *p* < .01, and *** *p* < .001). Kruskal-Wallis test, followed by a post-hoc Dunn test.

We then assessed the functional differences between the three GMC types. We focused on carbohydrate metabolism, particularly that mediated by glycoside hydrolases (GH), and features relating to pathogen carriage. GH repertoire profiling showed that GH13, GH2, GH77, GH3, and GH20, which target both fiber and milk carbohydrates, were the most abundant in the gut microbiome, regardless of GMC type (Figure 5a, Table S1). Multiple GHs differed in abundance between the three GMC types (Figure 5a, Table S3). Specifically, GMC type 1 differed from the other two types in terms of the abundance of several GHs related to host glycan metabolism: GH29 (fucosidase), GH33 (sialidase), and GH112 (GNB/LNB phosphorylase) (Figure 5a, Figure S3B, and Table S3). We then investigated the differences between GMC types in terms of the abundance of genes encoding pathogenic factors with Pathofact, a pipeline that can predict functions related to pathogens from metagenomic data. We found that the relative abundance of antimicrobial resistance genes, virulence factors, and toxins was higher in GMC type 3 than in the other GMC types (Kruskal-Wallis test, followed by a post-hoc Dunn test, *p* < .001 (Figure 5b). Overall, our findings reveal differences in both the composition and functional features between community types for the gut microbiomes of infants from rural Kenya.

### Maternal human milk phenotype and HMO composition

**Figure 6.**
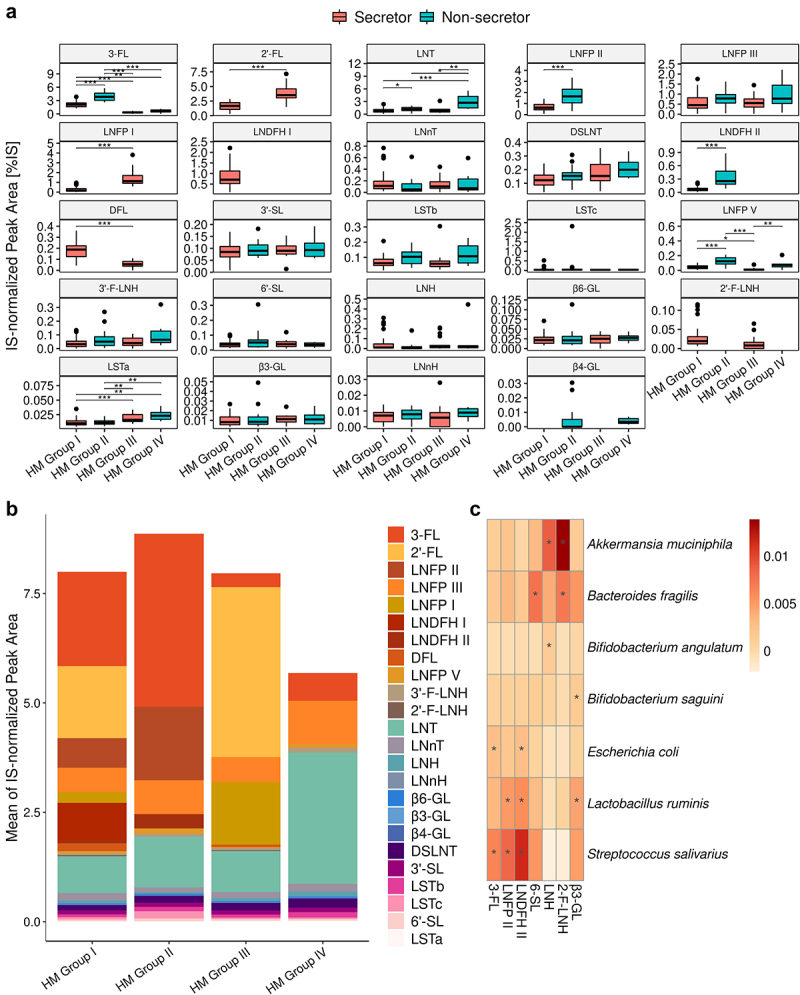
Abundance of the major HMOs stratified by maternal HM group. A. Boxplot of HMOs ranked in descending order of abundance (Mann-Whitney test, * FDR <0.05, ** FDR <0.01, and *** FDR <0.001). B. Mean proportion of HMOs in the different HM groups. Neutral fucosylated HMOs are shown as an orange-red gradient, neutral non-fucosylated HMOs are shown as a blue-green gradient and sialylated acids are shown as a pink-purple gradient C. Heatmap of MaAsLin2 correlation coefficients for the relationship between HMO abundance and bacterial species. HMOs are shown in descending order of abundance.

We then investigated the HMO profiles (based on relative abundances) of 90 HM samples. In total, 250 valid peaks (equivalent to at least as many different HMOs) were detected in at least one of the 90 HM samples. We focused on 24 HMOs accounting for about 80% of total HMO content: 2’- fucosyllactose (2’-FL), 3-fucosyllactose (3-FL); 2’- and 3’-fucosyllacto-N-hexaose (2’- and 3’-F-LNH); 3’- and 6’-sialyllactose (3’- and 6’-SL); difucosyllactose (DFL); disialyllacto-N-tetraose (DSLNT); LNFP I, LNFP II, LNFP III, and LNFP V; LNDFH I and LNDFH II; lacto-N-hexaose (LNH); lacto-N-neohexaose (LNnH); lacto-neotetraose (LNnT); lacto-tetraose (LNT); sialyllacto-N-tetraose (LST)a, LSTb, and LSTc; β1-3’-, β1-4’-, and β1-6’-galactosyllactose (β3-, β4-, and β6-GL) (Figure 6). The least prevalent HMOs were β4-GL (present in 17% of all donors, but in 57% of all secretor-negative HM samples). The abundance of LNDFH II was low in all secretor HM samples with values above the LOQ. Maternal secretor and Lewis (SeLe) phenotype or HM group was assigned based on the presence of specific fucosylated HMOs. HM typing showed that 48.9% of the HM samples corresponded to HM group I (Se^+^, Le^+^), 22.2% to HM group II (Se^−^, Le^+^), 22.2% to HM group III (Se^+^, Le^−^) and 6.7% to HM group IV (Se^−^, Le^−^). After grouping by secretor and Lewis status, 71% of the mothers were found to be secretors (HM groups I and III) and 29% were non-secretors (HM groups II and IV). Similarly, 71% of the mothers were Lewis-positive (HM groups I and II) and 29% were Lewis-negative (HM groups III and IV); the prevalence of Lewis positivity was higher in this population than in a European population studied in more detail.^41^ The most abundant HMOs in all 90 HM samples, regardless of Se or Le status, were 3-FL, LNT, and LNFP III. The abundance of 3-FL, LNT, LNFP II, LNFP III, LNFP V, LSTb, and LNDFH II was higher in milk from non-secretors than in milk from secretors (Mann-Whitney test, FDR<0.05) (Figure S4). More detailed studies of the four HM groups showed that 3-FL and LNFP V were more abundant in HM of group II (Se-, Le+), LNFP I and 2’-FL were more abundant in HM of group III (Se+, Le-), and LNT was more abundant in HM of group IV (Se-, Le-) (Figure 6a and b). Overall, in this cohort of infants with prolonged breastfeeding, the occurrence and abundance of HMOs differed between maternal HM phenotypes.

### Association of HMO profiles and HM groups with the infant gut microbiota

Previous studies have investigated the association between maternal HMOs, HM phenotype and the infant gut microbiota in different populations, and at various stages of lactation. We first investigated the association of individual HMOs with the gut microbiome in a linear mixed model analysis based on the 20 most abundant bacterial species (Table S4). Only positive correlations were retained after FDR adjustment (FDR <0.1) (Figure 6c), with *Streptococcus salivarius* having the largest number of significant correlations with fucosylated HMOs such as 3-FL, LNFP II, and LNDFH II. We then investigated the association between HM secretor status and the infant gut microbiome. The abundances of multiple bacterial species (including *Escherichia coli, Streptococcus salivarius, Bifidobacterium saguini*, and *Bacteroides thetaiotaomicron*) were higher in the gut microbiota of the infants of non-secretor mothers, whereas the gut microbiota of the infants of secretor mothers was enriched in *Bifidobacterium pullorum* and *Clostridium saccharolyticum* (Table S5) (DESeq2, FDR<0.05, age as a covariate). No difference in the overall gut microbiota (alpha- and beta-diversity), or in the prevalence of gut microbiota community types was observed (Figure S5 A-C).

Finally, given the higher prevalence of HM group III than in most previous studies, we explored the possible differential association of this HM group with the gut microbiome. The abundance of *Bifidobacterium pseudocatenulatum* was higher and that of *Klebsiella pneumoniae* was lower in HM group III than in HM groups I and II, respectively (DESeq2, FDR<0.05) (Figure S6A), with no overall difference in the global gut microbiota (Figure S6 B and C). There was a trend of lower pH in HM group III compared to HM group I (Mann-Whitney, p = .06). A network analysis of bacterial species of the three major HM groups showed differential co-variation between bacterial species including *Bifidobacterium* (Figure S7).

Our results indicate that associations between HMOs and the gut microbiota may be population-specific.

## Discussion

During early life, the gut microbiota is highly dynamic, responds to the environment and is crucial for health. In this study, we used metagenomics to explore the variability and function of the gut microbiome in an understudied population, 6 to 11-month-old infants living in rural Kenya. We found that the gut microbiome of the infants studied here were enriched in *B. longum* and, specifically, *B. longum* subsp. *infantis*. Several gut microbiota community types were identified that differed in composition and functional features.

In previous studies, *Bifidobacterium* was found to be the most abundant genus in the gut microbiota in six- to nine-month-old Kenyan infants.^28,31,32^ Here, using shotgun metagenomics, we showed that the gut microbiome of breastfed Kenyan infants also receiving complementary food is dominated by *B. longum* species, consistent with the findings of other studies of infants of similar age living in rural environments in Malawi,^42,43^ and Indonesia.^8^

This indicates that there is a sustained enrichment in bacteria from the *Bifidobacterium* community in partially breastfed infants. *B. breve, B. bifidum*, and *B. kashiwanohense* were the other most abundant *Bifidobacterium* species; these species are known to metabolize HMOs, albeit with different efficiencies.^44–47^ Network analysis identified *B. kashiwanohense* as key to the interaction between two *Bifidobacterium* subnetworks, suggesting a possible ecological role in shaping *Bifidobacterium* community despite the lower abundance and narrower range of HMOs metabolized by this species relative to *B. longum, B. breve*, and *B. bifidum*, which have a wider metabolic range.^44,45^ We further differentiated between *B. longum* subsp. *longum* and *B. longum* subsp. *infantis* in a pangenomic analysis of the *B. longum* species. We focused on an HMO cluster of genes encoding proteins involved in both the import and metabolism of HMOs^12,48^ and genes encoding proteins involved in arabinose metabolism.^34^ The prevalence of *B. infantis* was high in metagenomes from Kenyan infants (> 80%), contrasting with its much lower levels in a cohort of mostly exclusively breastfed four-month-old Swedish infants (20%),^35^ in which *B. longum* subsp. *longum* was the more abundant of the two subspecies. Our results are consistent with studies reporting a higher prevalence of *B. infantis*, typically reaching more than 70%, in infants from countries with limited resources, such as Gambia (76.9%) and Bangladesh (86%),^7^ or from rural settings in the USA.^10^ The prevalence of this subspecies is lower (<20%) in more developed countries with higher rates of immune disorders,^11,12,49^ as recently shown in a global meta-analysis.^13^ We then studied the variation of the gut microbiome among the infants of our study cohort. We identified GMC types by the Dirichlet multimodal mixture method,^50^ a clustering approach widely used in studies of the gut microbiome, including investigations in infants.^37,38^ Previous studies have shown that GMC type is influenced by age, feeding and geographic location.^8,11,12,51,52^ Here, in a cohort with a narrow range of age and feeding practices, we found differences in alpha-diversity between GMC types, the least diverse of which was enriched in *Bifidobacterium*, whereas the most diverse was enriched in multiple genera including Enterobacteriaceae. The GMC type with the highest abundance of *B. longum* and in which *B. infantis* and *B. breve* were most frequently exclusively detected had the lowest pH and alpha-diversity, and a higher abundance of some glycoside hydrolases involved in host/animal glycan metabolism, possibly reflecting the metabolism of HMOs and/or mucosal glycans.^53^ Analysis of Metagenomes-assembled genomes would allow to compare the diversity and function of gut microbiome across populations in early life.^54^ Furthermore, the frequency of genes encoding antimicrobial resistance, virulence factors and toxins was lower in GMC type 1, consistent with the lower abundance of Enterobacteriaceae species and a lower pH than for the other two GMC types. Fecal pH was generally low in this study (mean of 5.3), consistent with the high levels of *Bifidobacterium* (specifically *B. infantis* and *B. breve*), reflecting the production of organic acids, such as lactic acid and acetic acid.^55^ In Western infants, fecal pH typically exceeds 6, and the abundance of *Bifidobacterium* is lower.^56^ The least prevalent GMC (GMC type 3) had the highest alpha-diversity and displayed the greatest enrichment in genera other than *Bifidobacterium*, regardless of age and calprotectin levels, a surrogate marker of inflammation. Diet is considered as a major determinant of gut microbiome composition and function. A previous study in infants showed that the cessation of breastfeeding, rather than the introduction of other foods, drove maturation of gut microbiome.^35^ In our study, most of the infants received cereals in addition to human milk. While this study provides a first insight into the variation of the gut microbiome in infants from rural Kenya, larger studies, with detailed and quantified recording of dietary habits, are now required.

We assessed the association with HMOs further, as most infants were still partially breastfed. Several factors, including genetic background and lactation stage, are known to affect HMO profile.^18^ The composition of HMOs in this study cohort was consistent with previous studies performed during the late lactation stage (> 6 months), as 3-FL levels have been shown to increase during the course of lactation.^41,57^ The association between individual HMOs, secretor status and the gut microbiome varies considerably between studies.^22–28^ We found several associations between individual HMOs and bacterial species, including known HMO-metabolizing species from the genera *Akkermansia, Bifidobacterium, Bacteroides*, and *Streptococcus*. The lack of association between abundant *Bifidobacterium* species *(B. longum, B breve and B. bifidum)* and individual HMOs, despite the observed enrichment in *Bifidobacterium*, may reflect metabolic cooperation between species, as observed in studies performed *in vitro*.^46,58^ We further evaluated the association of maternal HMO secretor status, with functional α-1-2-fucosyltransferase (FUT2) status, HMO abundance and infant gut microbiome. In our study, 71% of the mothers were secretors, which is in the range with other studies from different populations as well as in African countries such as South Africa, Gambia, Ghana, Ethiopia, and Malawi.^21,59^ Stratification into four HM groups based on Se/Le genes revealed a higher prevalence of Lewis-negative samples, belonging to HM group III (22.2%) in particular in line with a recent study,^60^ than in most other populations studied.^29,57^ 2’-FL, a HMO widely studied due to its effects on the gut microbiome, such as the stimulation of *Bifidobacterium* and the inhibition of pathogens,^61,62^ was more abundant in HM group III, consistent with previous studies.^29,57^ Despite the limited sample size of this study, the abundance of *B. pseudocatenulatum* was higher, and that of *Klebsiella* spp. lower, in HM group III. Interestingly, Newburg et al. reported that infants fed with HM group III were significantly less likely to be infected with a pathogenic *E. coli*^63^ potentially suggesting a specific association between HMOs and gut microbiome in our cohort, and potentially rural African population. Further larger studies are required to confirm these differences in abundances between HM groups and, specifically, to determine whether the higher prevalence of HM group III resulted from the evolutionary pressure imposed by pathogens in rural regions like those studied here in Kenya.^28,41,57^

Overall, this study provides new insight into the composition and function of the gut microbiota of infants from rural Kenya, and the variability of genetic factors and HMO profiles. The enrichment of the gut microbiome in specific species of *Bifidobacterium* is associated with functional differences such as a low pH, and low frequencies of antimicrobial resistance genes and virulence factors. Our study also highlights the existence of variable patterns, with some infants harboring a higher abundance of pathogens, which may be useful to guide microbiome-based nutritional interventions. The association between the baseline gut microbiota and clinical responses (iron absorption) to nutritional intervention (prebiotics) would also be of considerable interest in this study cohort. In addition, larger and longitudinal studies in rural Africa should improve our understanding of the structural and functional variation of the gut microbiome of African infants, and in generally understudied populations, shedding light on the contribution of intestinal symbionts to health.

## Materials and methods

### Study design and participants

We studied a subset of infants (*n* = 105) enrolled in a single-blind, randomized controlled intervention trial with three arms, conducted in Msambweni and the surrounding rural communities in Kwale County on the southern coast of Kenya. The study was conducted from July 2019 (rainy season) to January 2020 (dry season). We enrolled 6 to 11-month-old infants with no reported current acute or chronic illness, and z-scores for weight-for-age and weight-for-length ≥-3. Infants were excluded if they were severely anemic (Hb <70 g/L), had regularly been given iron-containing mineral and vitamin supplements within the last two months, or had received antibiotics in the month before study enrollment.

### Stool collection and DNA extraction

We analyzed fecal samples from 105 infants. The caregivers were asked to collect the fecal samples carefully from the infant on the evening before or the morning of the study visit. They were provided with: i) a specific tube (OMNIgene GUT, OM-200, DNAgenotek, Canada) for the analysis of gut microbiota profile; and ii) polystyrene stool tubes (Sarstedt, Sevelen, Switzerland) for the determination of fecal calprotectin levels and fecal pH. Fecal samples were split into aliquots and frozen at −20°C. All aliquots were stored at the study site until shipment on dry ice to the ETH Zurich, Switzerland, for further analyses. Fecal calprotectin was determined with an ELISA kit (Eurospital, Italy) and fecal pH was determined with a digital pH meter (Metrohm, Switzerland). For fecal DNA extraction, samples were vortexed for 60s and incubated at 50°C for 30 min (as recommended by OMNIgene.GUT for viscous samples). The pellet obtained after centrifugation (1 min at 13. 800 g) was incubated with 200 μL enzyme cocktail (50 mg/mL lysozyme, 20 U/mL lysostaphin and 150 U/mL mutanolysin, all from Sigma-Aldrich, St. Louis, USA), at 37°C for 30 min before mechanical disruption with a FastPrep‐24™ instrument (MP Biomedicals™) set at speed 6.0, for two 60-second periods. DNA was isolated with the QIAamp®PowerFecal®Pro DNA-kit (QIAGEN®, Hilden, Germany) according to the manufacturer’s instructions. DNA was eluted in a final volume of 75 μL. The DNA preparation was subjected to quality control by spectrophotometry on a NanoDrop™ 2000c spectrophotometer (Thermo Fisher Scientific, Waltham, USA), according to the manufacturer’s instructions.

**Metagenomic shotgun sequencing and preprocessing**. In total, 105 samples were analyzed by Shotgun metagenomics at ADM-Biopolis (Paterna, Spain). DNA was quantified fluorometrically with a Qubit Fluorometer (Themo Fisher Scientific, Carlsbad, USA). Sequencing libraries were prepared with the Nextera XT DNA sample preparation kit, according to the manufacturer’s instructions. Samples were sequenced on a NovaSeq 6000 platform with the kit for 151 bp paired-end reads, resulting in 34.7 million (± 7.8 million) paired-end reads per sample. Demultiplexed reads were filtered with BBTools^64^ and quality filtering was performed with NGLess v1.0.0-Linux64 software.^65^ Reads were filtered for 97% identity to the human genome (hg19), to obtain Q20 reads with a minimal length of 45 nt and no trace of human DNA contamination. After filtering, a mean of 26.6 million (± 5.6 million) paired-end reads per sample were retained.

### Genomic assembly and annotation

Sequences were assembled with MetaSPADES genome assembler v3.13.0 (Nurk et al., 2017), with a range of *k*-mer sizes (21–127) and the assemblies were filtered to exclude sequences of less than 500 bp in length. Assembly performance was analyzed with QUAST v.5.0.0,^66^ using the default parameters. Open reading frames (ORFs) were predicted with Prodigal v2.6.3^67^ in metagenomic mode and then filtered to obtain ORFs with start or stop codons, yielding a mean of 53.085 (± 24.138) genes per sample. Genes from all samples were clustered with CD-HIT v4.8.1.^68^ with the following criteria: 90% alignment coverage and 95% gene sequence identity, to generate a non-redundant *de novo* gene set. A count matrix for each sample was generated from the non-redundant *de novo* gene set with NGLess v.1.0.0,^65^ retaining only primary mapped reads with a minimum match size of 45 nt displaying at least 95% alignment, with a dist1 for multiple mapping reads. Pathogenic factors were identified with PathoFact software, using default parameters.^69^ Carbohydrate-active enzyme (CAZy) was assessed with dbcan2.^70^ In short, a triple annotation was performed with (i) HMMer against the dbCAN HMM database, (ii) DIAMOND search against the CAZy pre-annotated CAZyme sequence database, and (iii) eCAMI run against the CAZyme database; CAZy terms annotated with at least two methods were retained for downstream analysis.

For taxonomic analysis, the filtered reads were aligned with single-copy marker genes present in almost all bacteria, viruses, and archaea. The relative abundances of the taxa identified were calculated with the MetaPhlAn3 v.3 pipeline.^71^ The pipeline used is depicted in Figure S1. *Bifidobacterium longum* subsp. *infantis* (*B. infantis*) and *B. longum* subsp. *longum* were detected with PanPhlAn software^72^ applied to the *B. longum* pangenome (14 *B. infantis* and 30 *B. longum* reference genomes) (Table S2). In short, *B. infantis* HMO cluster genes (Blon_2331–Blon_2361) were selected from the *B. longum* (species) pangenome (Sela et al., 2008), together with the *B. longum* subsp. *longum-*specific genes (*araA* and *araD*),^34^ to assess the presence of these subspecies in the metagenomes for Kenyan and Swedish infants. We excluded 18 samples from Swedish infants (18% of the sample set) and two samples (2% of the sample set) from Kenyan infants from the analysis (below the default PanPhlAn threshold for the presence of *B. longum* genes).

**External dataset**. A four-month-old infant microbiome gene set catalog was obtained from the GIGAdb website (gigadb.org/dataset/100145)^35^ and compared with the non-redundant *de novo* gene set.

### Human milk collection and HMO profiling

Human milk samples for HMO analysis were obtained by manual milk expression by the mother into a clean plastic container. The samples were kept cool until homogenization by the study team. They were then split into 1–2 mL portions and stored at −20°C. All aliquots were stored at −20°C at the study site until shipment on dry ice to the ETH Zurich, Switzerland. For the HMO composition analysis reported here, the HM samples were transported on dry ice to the glycoanalytical laboratory (glyXera GmbH, Magdeburg, Germany). The qualitative and quantitative HMO composition of each individual HM sample was determined with the glyXboxCE™ system (glyXera GmbH, Magdeburg, Germany) based on multiplexed capillary gel electrophoresis with laser-induced fluorescence detection (xCGE-LIF).^73^ In accordance with the glyXera GmbH kit protocol (KIT-glyX-OS.P-APTS, glyXera GmbH, Magdeburg, Germany), the pure HM samples were diluted 1:100, spiked with an internal standard (IS) (oligosaccharide (OS) quantification standard solution, OS-A5-N-1 mL-01; part of the KIT-glyX-Quant-DP5, all from glyXera GmbH, Magdeburg, Germany) and treated with a denaturation solution. The free OS were labeled with 8-aminopyrene-1,3,6-trisulfonic acid (APTS), purified and determined with the glyXbox™ system. All measurements included the addition of a migration time alignment standard (glyXalign4; STD-glyXalign-4-S, glyXera GmbH) to the sample. Finally, glyXtoolGUI™ software (Beta v0.8.11, glyXera GmbH, Magdeburg, Germany) was used for the processing and analysis of the *HMO Fingerprints* data (normalized electropherograms). The limit of quantification (LOQ) was determined from the signal-to-noise ratio (SNR) of each *HMO Fingerprint* calculated as described by Ullsten et al.^74^ The LOQ was defined as an SNR of 10 and the limit of detection (LOD) was defined as an SNR of 3. The respective noise for each sample was determined after migration time alignment of the unsmoothed data in the late migration time range (approximation range = degree of polymerization (DP) 18< DP<20). Peaks with intensities below the LOQ but above the LOD were picked. All peaks ≥LOQ were considered and their IS-normalized peak areas were calculated (as percentages relative to the peak area of the IS [% IS] (= nPA)). All peaks ≥LOD but <LOQ were replaced with a fixed value; the peaks followed a triangular distribution, so the respective peak areas were replaced with LOQ/√2.^75^ All HM samples were assigned to a maternal secretor and Lewis (Se/Le) phenotype (HM groups I–IV) based on the presence or absence of specific α1-2- and/or α1-4-fucosylated HMOs, as previously described.^73^ The assignment of maternal secretor status was based on the presence of 2’-fucosyllactose (2’-FL), difucosyllactose (DFL), and lacto-N-fucopentaose (LNFP) I, and the determination of Lewis status was based on the presence of LNFP II and lacto-N-difucohexaose (LNDFH) II. Differences in HMO abundance between maternal secretor status and HM types were assessed with Mann-Whitney tests or Kruskal-Wallis tests followed by post-hoc Dunn’s test, respectively, with adjustment for false discovery rate (FDR) by the Benjamini-Hochberg mechanism (FDR<0.05).

### Gut microbiota statistical analysis

All analyses were performed with R version 3.6.0. The R package Vegan (function *richness*) was used to calculate alpha-diversity metrics (Shannon’s H, inverse Simpson’s index, and feature richness). Samples were rarefied with the phyloseq library in R (rarefy_even_depth function). Bray-Curtis dissimilarity was used for the analysis of beta-diversity. Permutational testing of variance (PERMANOVA) between groups was performed with the adonis2 function (R package Vegan). For the identification of gut microbiota community types (GMC types) at genus level (relative abundance), we used the Dirichlet multinomial mixture (DMM) approach^50^ with Laplace number selection. Kruskal-Wallis tests were performed, followed by post-hoc Dunn’s tests, for comparisons between GMC types for the parameters studied (CAZy, main genera and species, age, fecal pH, and fecal calprotectin level, alpha-diversity, and ARG) followed by FDR correction by the Benjamini-Hochberg method for CAZy, main genera, and species. A Pearson’s Chi-squared test was used to determine whether GMC types were associated with secretor status. Network analysis was performed by center log ratio (CLR) normalization and SparCC correlation with the NetCoMi R package, based on the top 20 species with a prevalence of at least 25%, and a correlation threshold of 0.2. We used DESeq2 package (version 1.26.0) to identify species with differential abundances, and different CAZy levels between secretors and non-secretors, with age as a covariate (count sums > 5, and present in at least five subjects). Significant fold-change differences between groups were evaluated with the negative binomial model-based Wald test implemented in DESeq2 (alpha risk = 0.05). A linear mixed model analysis was performed to analyze the relationships between the 20 most abundant bacterial species and 1) calprotectin level, 2) pH, and 3) the abundance of individual HMOs, in MaAsLin2 with default settings (v.1.0.0).^76^ Age was considered as a random effect and individual HMO, pH and calprotectin levels were considered as fixed effects. Heatmaps based on the correlation coefficients (effect size) with FDR <0.1 (Benjamini-Hochberg) or *p*< .1 were generated for individual variables (pH, calprotectin, and HMOs).

## Supplementary Material

Supplemental Material

## Data Availability

Metagenomic sequences associated with this project were deposited in EMBL under BioProject accession no. PRJEB52748. The source codes used in this study are available from GitHub (github.com/danone/Kenya.study)
